# Comparative Transcriptome Analyses Characterize Expression Signatures Among Males, Females, Neo-Males, and Gynogenetic Females in the Yellow Drum (*Nibea albiflora*)

**DOI:** 10.3389/fgene.2022.872815

**Published:** 2022-05-13

**Authors:** Qihui Zhu, Zeqian Qin, Ruiyi Chen, Ligai Wang, Peng Tan, Dongdong Xu

**Affiliations:** ^1^ Key Lab of Mariculture and Enhancement of Zhejiang Province, Zhejiang Marine Fisheries Research Institute, Zhoushan, China; ^2^ School of Fisheries, Zhejiang Ocean University, Zhoushan, China

**Keywords:** RNA-seq, histological observation, gynogenesis, neo-male, brain, gonad, yellow drum

## Abstract

The yellow drum (*Nibea albiflora*) is one of the most important marine economic fish in China, and its sexually dimorphic growth makes it preferable for mono-sex culture. Although gynogenesis and neo-male induction techniques have been established, the molecular pathways and regulatory mechanisms of sex determination and maintenance in gynogenetic females and neo-males remains far from fully understood. In this study, the gene expression profiles were investigated in the gonads and brains of wild-type male, wild-type female, neo-male, and gynogenetic female yellow drum using comparative transcriptome analyses. Generally, a total of 52,999 novel transcripts were obtained in RNA-seq, of which 45,651 were isoforms of known protein-coding genes, 1,358 novel protein-coding genes, and 5,990 long non-coding RNAs. We found that the differences between wild-type males and neo-males and between wild-type females and gynogenetic females were relatively small at both the histological and transcriptomic levels, indicating that artificial gynogenesis or hormonal sex reversal may have minimal effects on normal female or male life function, respectively. In the brain, pathways such as “Oocyte meiosis”, “Cell cycle”, and “Riboflavin metabolism” were found to be significantly enriched. In the gonads, pathways such as “Prolactin signaling pathway”, “PPAR signaling pathway”, “Cholesterol metabolism”, and “Jak-STAT signaling pathway” were found to play important roles in maintaining the regular proliferation and differentiation of females and males in yellow drum. In particular, we found that *zp4* might be an effective molecular marker to differentiate between gynogenetic and normal females owing to its unique expression pattern. The results of this study may help to elucidate the molecular mechanisms involved in sex maintenance in the gonads and brain and provide basic data for genetic breeding of the yellow drum.

## 1 Introduction

Sex dimorphism, especially size and morphological dimorphism, is quite common in fish. One gender may mature later than the other, allowing nutrients to translate into ontogenesis and weight gain during maturation, resulting in a larger body size compared to that of the opposite gender. Therefore, it is effectively used in fish aquaculture to increase yield through sex control. Artificial gynogenesis and hormonal sex reversal techniques have been proven to be effective methods and are widely used in fish breeding. The yellow drum (*Nibea albiflora*) is a sciaenid of great commercial value in East Asia, which exhibits significant sexually dimorphic growth, with females growing 30% faster than males (Xu et al., 2010). Since a mono-sex (all female) culture could potentially enhance the efficiency and profitability of its aquaculture industry, various laboratories, including ours, have already developed an effective protocol to induce meiotic gynogenesis (Chen et al., 2017) and the protocol for neo-male (genetic female) induction using hormone treatment, as well as the subsequent protocol for producing an all-female population using neo-males and gynogenetic females (Xu et al., 2018). The critical period of sex differentiation in yellow drum has been detected via histological observation, which occurs around 36 days post hatching with a total length about 21.20 ± 3.90 mm (Lou et al., 2016). A male-specific genetic marker was detected and a PCR-based method for genetic sex identification was established accordingly (Sun et al., 2018), which made it possible to validate the mono-sex population. Moreover, the whole genome sequence of the yellow drum has been assembled, and this helped to elucidate the genetic mechanisms of its biological characteristics (Han et al., 2019; Xu et al., 2021). Nevertheless, to date, little is known about the molecular differences between gynogenetic and wild-type female or neo-male and wild-type male yellow drum.

Sex reversal through hormone treatment, in which sexually undifferentiated fish are exposed to exogenous sex steroid with an appropriate dosage and duration, has been conducted in many fishes (Kobayashi and Iwamatsu, 2005; Lee et al., 2009; Xu et al., 2018). The success of sex reversal could be attributed to the activation of steroidogenic pathways during gonad differentiation (Martinez et al., 2014). Although the processes of sex determination and sex differentiation are very complex, as a product of the *cyp19a1a* gene, the role of estrogen and the aromatase enzyme complex is considered to be a well-conserved factor in teleost sex differentiation (Devlin and Nagahama, 2002). It has been reported that *cyp19a1a* is upregulated to induce ovarian differentiation, while its expression is blocked in males to prevent estrogen production (Zhang et al., 2014; Baroiller and D'Cotta, 2016). Accordingly, in male vertebrates, gene *dmrt1* is reported to play a key role in testicular development (Herpin and Schartl, 2011). Many studies have also investigated brain’s role during gonadal development (Dennis, 2004; Lee et al., 2018; Zou et al., 2020), and suggested that the brain could also affect gonadal sex. For example, in the Japanese quail (*Coturnix japonica*), testis function is disrupted by forebrain transplants prior to sexual differentiation in male and female embryos (Gahr, 2003). To date, the molecular mechanisms underlying sexual differentiation of the brain remain to be thoroughly investigated. Therefore, identification of sex-related genes in the gonads and brains of gynogenetic and wild-type females, and neo-males and wild-type males, respectively, may help to elucidate the gene interaction between the gonads and brain, as well as the molecular regulatory mechanisms of sex reversal.

RNA sequencing (RNA-seq) has been an effective method for exploring gene expression levels, and gonadal transcriptomic studies have been conducted in several fish species. For example, in the channel catfish (*Ictalurus punctatus*), male-related genes including *cxcl12* and *gsdf,* were screened though transcriptomic comparison of the testes and ovaries (Zeng et al., 2016). Various of genes associated with gonadal development and gametogenesis, such as *bmp15*, *nanos3*, and *amh*, were identified via transcriptomic analysis in the spotted knifejaw (*Oplegnathus punctatus*), and these genes were considered to play important roles in germ line cell maintenance of the gonads and gonadal physiology regulation (Du et al., 2017). Similarly, genes potentially involved in reproduction and gonadal development were identified in spotted scat (*Scatophagus argus*) *via* comparative transcriptome analysis, such as male-biased *dmrt1*, *amh*, *gsdf*, *wt1a*, *sox9b*, and *nanos2*, and female-biased *foxl2*, *gdf9*, *bmp15*, *sox3*, *zar1*, and *figla* (He et al., 2019). Nevertheless, the expression profiles of gynogenetic females and neo-males were seldom investigated on transcriptomic levels.

In this study, the gonads and brains from four types of yellow drum (wild-type males, wild-type females, gynogenetic females, and neo-males) were subjected to comparative transcriptional analyses to reveal the differences among them at the transcriptomic level. The results of this study may provide new insights into the molecular mechanisms involved in sex dimorphism, sex maintenance, and sex reversal in the gonads and brain, as well as elucidate the genetic basis behind sex control breeding, in the yellow drum.

## 2 Materials and Methods

### 2.1 Ethics Statement

The collection and handling processes for all animals used in this study were approved by the Animal Care and Use Committee of the Marine Fisheries Research Institute of Zhejiang Province and Zhejiang Ocean University. All experimental procedures were performed according to the prescribed guidelines.

### 2.2 Experimental Fish

The fish used in this study were reared at and collected from the hatchery of the Marine Fishery Institute of Zhejiang Province, Xixuan Island, Zhoushan, China. According to previous studies, the gynogenetic females were induced by cold shock at 4°C for 8 min after fertilization with genetically inactivated heterologous spermatozoa (Chen et al., 2017), and the neo-males were induced by 17α-methyltestosterone immersion for 2 h/day from 30 to 90 days post hatch (dph) (Xu et al., 2018). Briefly, 10 individuals each from the control group (95.28 ± 14.37 g), gynogenetic group (94.37 ± 15.20 g), and neo-male group (95.12 ± 15.32 g) were randomly sampled at 180 dph, and the sample individuals were dissected immediately to retrieve their brains and gonads. The brain samples were snap frozen in liquid nitrogen immediately and stored at −80°C for RNA isolation, and each gonad sample was divided into two aliquots: one was snap frozen stored in liquid nitrogen and stored at −80°C, and the other was fixed with Bouin’s solution for histological analysis. After sex identification by histological observation of the gonad, three samples each from both male and female of the control group, female of the gynogenetic group and male of the neo-male group, were randomly chosen for RNA isolation and subsequent analyses.

### 2.3 Histological Observation

Gonadal samples fixed by Bouin’s solution were sliced into 5 μM-thick sections using standard paraffin embedding methods, followed by staining with hematoxylin and eosin. Then a microscope (Axio Imager A2; Zeiss, Oberkochen, Germany) equipped with a digital camera (Axiocam 506; Zeiss) was used for histological observation of the stained samples. And therefore the phenotypic sex of each fish was identified.

### 2.4 Total RNA Extraction and Illumina Sequencing

The brain and gonad samples of three fish from each group (24 samples in total), were used for transcriptomic analysis. Total RNA was isolated by the TRIzol reagent (Invitrogen, Waltham, MA, United States). The RNA integrity was evaluated by Agilent 2,100 Bioanalyzer (Agilent Technologies, Santa Clara, CA, United States), and samples with RNA integrity numbers ≥ 7 were subjected to subsequent analyses. The sequencing libraries were constructed though TruSeq Stranded mRNA LT Sample Prep Kit (Illumina, San Diego, CA, United States), which were then sequenced on the Illumina HiSeq X Ten platform.

### 2.5 Transcriptomic Analysis and Functional Annotation

RNA-seq data were processed according to the procedures described in a previous study (Li et al., 2021), and clean reads were aligned (Langmead and Salzberg, 2012) and mapped to the *N. albiflora* genome (NCBI accession: PRJNA577200) for gene annotation. Gene functions were annotated the NCBI nonredundant (NR), SwissProt, and clusters of orthologous groups (COG) for eukaryotic orthologous groups (KOG) databases (Altschul et al., 1990). Then gene ontology (GO) classification was performed by mapping the relationship between SwissProt and GO terms. The Kyoto Encyclopedia of Genes and Genomes (KEGG) database was also used to annotate the potential underlying metabolic pathways of the genes (Kanehisa et al., 2008).

We obtained the fragments per kilobase of transcript per million fragments mapped (FPKM) (Trapnell et al., 2010) and the read count value of each gene (Roberts et al., 2013). Then the correlation coefficients between samples were calculated to demonstrate the reliability of the experiment and the rationality of sample selection. The differentially expressed genes (DEGs) were obtained though DESeq (2012) functions of “estimate Size Factors” and “nbinom Test” with an adjusted *p-*value ≤ 0.001 and foldChange ≥ 4 as the thresholds for significant differential expression, and the DEGs with FPKM ≤ 0.5 in both compared groups were removed. The GO enrichment and KEGG pathway enrichment analyses of the DEGs were conducted using R based on hypergeometric distribution under default parameters, and the thresholds of significant enrichment was set at *q* value < 0.05. For DEG analyses between different groups, Venn diagrams were generated *via* the website tool: http://bioinformatics.psb.ugent.be/webtools/Venn/.

### 2.6 Quantitative Real-Time PCR Validation

Gonadal samples for transcriptomic analysis were also used for validation though quantitative real-time PCR (qPCR). The total RNA was extracted by Trizol Reagent (Invitrogen, Waltham, MA, United States), followed by reverse-transcription *via* a cDNA synthesis kit (DRR047A, Takara). The concentration and quality of the RNA were measured by absorbance at 260 nm and agarose gel electrophoresis, respectively.

A quantitative thermal cycler (ABI StepOnePlus; Thermo Fisher Scientific, Waltham, MA, United States) was used to perform qPCR. Using SYBR Green real-time PCR mix (DRR041A; Takara Bio, Dalian, China), qPCR was conducted following the protocol described in a previous study (Zhu et al., 2020). And qPCR was performed in triplicate for each sample. The gene *β-actin* was used as the nnternal reference according to previous studies on yellow drum (Xu et al., 2018; Song et al., 2019; Wang et al., 2019; Zhu et al., 2020). All primers for qPCR were designed *via* Primer Premier 6.0, and the detailed information on the primers is listed in Supplementary Table S2. Relative gene expression levels were evaluated using the 2^−ΔΔCT^ method (Livak and Schmittgen, 2001). The fold change between different groups of each gene was calculated in both qPCR and RNA-seq results, and the correlation coefficients were calculated accordingly using Microsoft Excel 2010.The qPCR results are presented as the mean ± standard error of the mean (SEM). Analysis of variance (ANOVA) and Tukey’s multiple comparison tests were conducted to compare the significant differences among the different groups using SPSS software (version 19.0; IBM, United States), where *p*-values < 0.05 were considered statistically significant.

## 3 Results

### 3.1 Histological Observation of the Gonads

**FIGURE 1:**
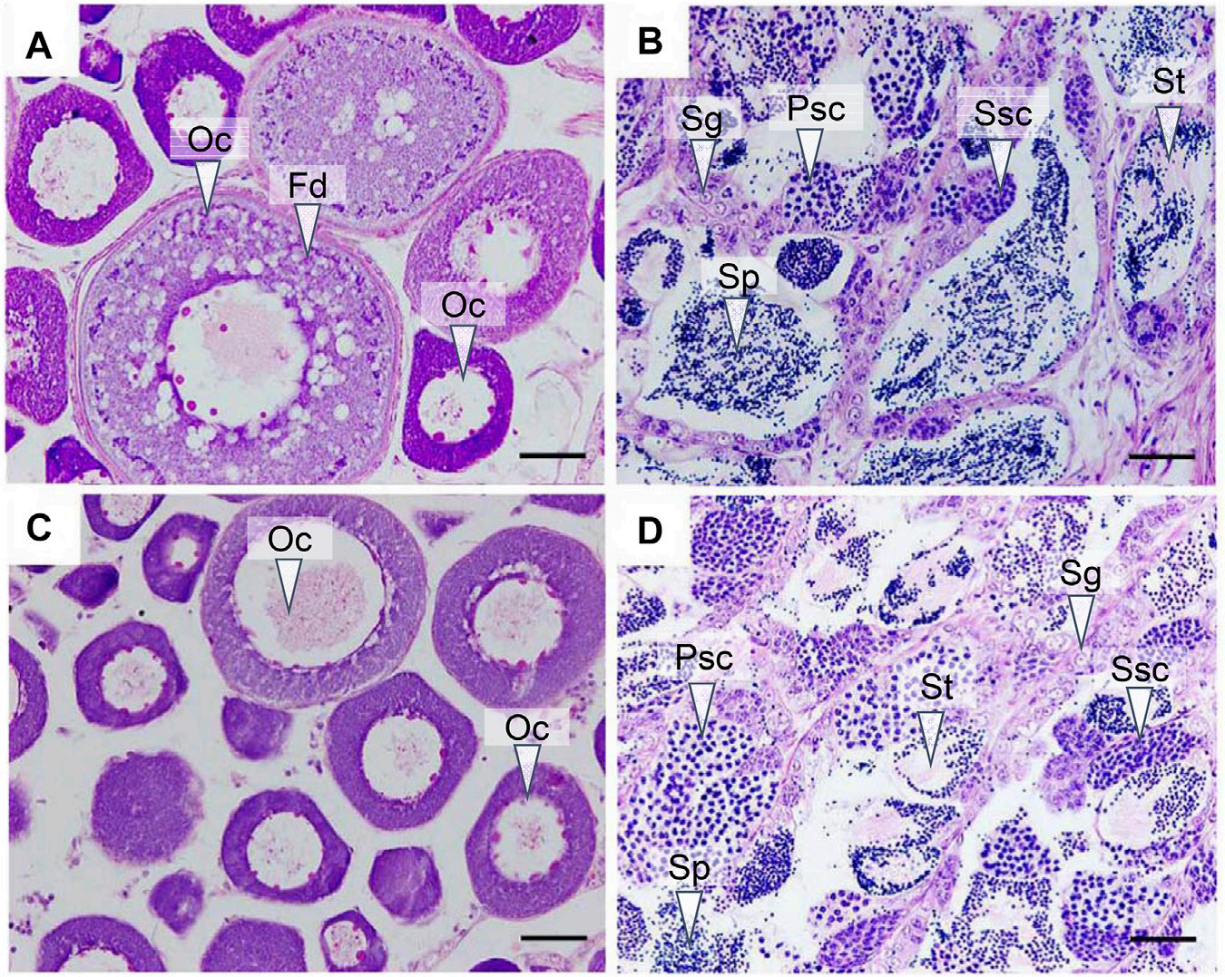
Histological observation of the gonads at 180 days post hatch (dph). **(A)** Ovary from the control group; **(B)** testis from the control group; **(C)** ovary from the gynogenesis group; and **(D)** testis from the neo-male group. Oc: oocyte; Fd: fat droplet; Sg: spermatogonia; Psc: primary spermatocyte; Ssc: secondary spermatocyte; St: spermatid; Sp: Sperm. Scale bars = 50 μM.

We first analyzed the gonadal development in yellow drum from different groups using histological staining. The ovary from the control group entered stage III at 180 dph, with layers of follicles in the cytoplasm and large fat droplets near the nucleus, illustrating the accumulation of nutrients in the oocytes (Figure 1A). The testis from the control group entered stage IV at 180 dph (Figure 1B), with different types of spermatogenetic cysts, such as spermatocytes and sperm cells, hinting at active spermatogenesis processes. The ovaries from the gynogenesis group and the testes from the neo-male group were also in stage III and stage IV, respectively, resembling the gonads from the control group (Figures 1C,D).

### 3.2 Transcriptome Data Analysis

**FIGURE 2:**
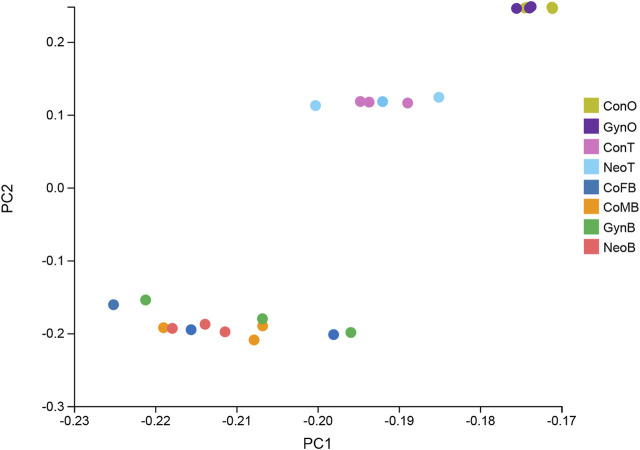
Principal component analysis result of the RNA sequencing (RNA-seq) samples.

**FIGURE 3:**
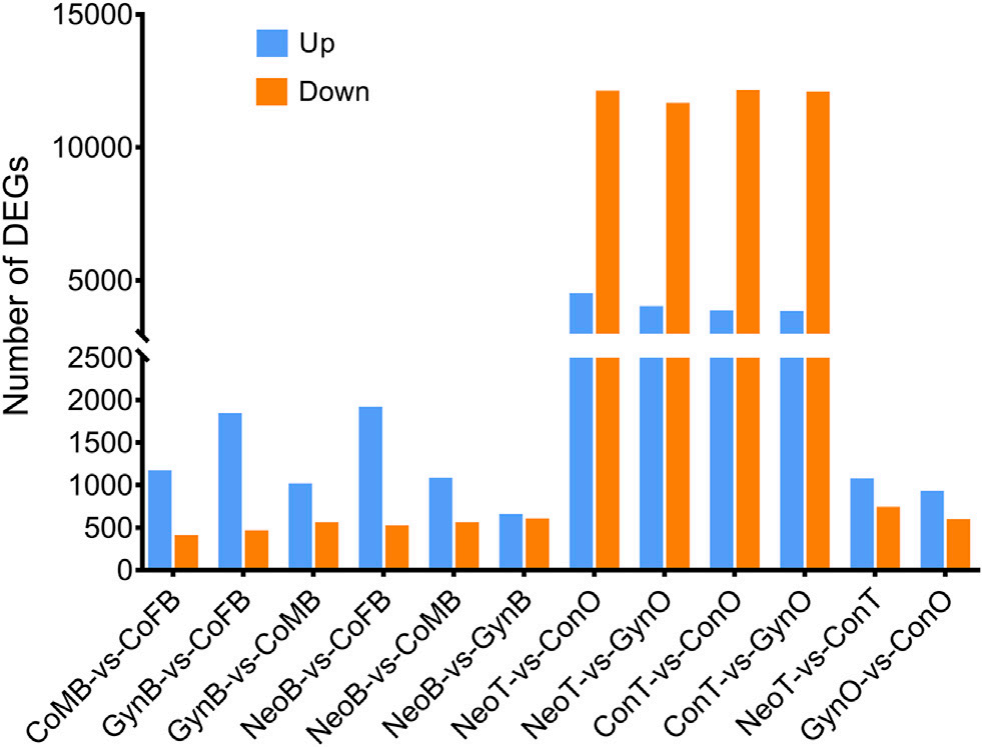
Comparison of differentially expressed gene (DEG) numbers.

A total of 24 samples were analyzed via RNA-seq, with three samples in each group, including female brains (CoFB), male brains (CoMB), ovaries (ConO) and testes (ConT) from the control group, brains (GynB) and ovaries (GynO) from the gynogenesis group, and brains (NeoB) and testes (NeoT) from the neo-male group. The reads data were deposited into the National Center for Biotechnology Information (NCBI) database under BioProject accession: PRJNA821274, and detailed information on the sequencing data and mapping are summarized in Supplementary Table S1. Clean reads were mapped on the annotated genome of *N. albiflora*, and an average of 86.96% of the clean reads could be successfully mapped, of which an average of 67.12% were uniquely mapped (Supplementary Table S1). A total of 52,999 novel transcripts were obtained, of which 45,651 were isoforms of known protein-coding genes, 1,358 novel protein-coding genes, and 5,990 long non-coding RNAs. The correlation coefficients between replicated samples were higher than 0.86; in fact, the correlation coefficients were good within the samples of the same type of tissue (Supplementary Figure S1). The principal component analysis results illustrated that the data were mainly distributed in three clusters: ovaries, testes, and brains, suggesting the good homogeneities among parallel samples, which also validated the results of the correlation coefficient analysis (Figure 2). The numbers of DEGs are shown in Figure 3. Generally, the number of DEGs from the testis-vs-ovary groups was larger than that of the brain-vs-brain, testis-vs- testis, or ovary-vs-ovary groups.

#### 3.2.1 Analysis of Differentially Expressed Genes from the Different Brain Groups

For the DEGs of the CoMB-vs-CoFB group, which represented the differences between wild-type male and female brains, 1,513 DEGs were involved. The GO terms “Structural constituent of cuticle” and “Structural constituent of cytoskeleton” were found to be significantly enriched, including five and nine genes, respectively. Further KEGG enrichment analysis showed that four pathways were significantly enriched, including “Oocyte meiosis”, “Cell cycle”, “Riboflavin metabolism”, and “Pathogenic *Escherichia coli* infection”. For the DEGs of the GynB-vs-CoFB group, which represented the differences between gynogenetic and female wild-type brains, respectively, 2,247 DEGs were involved. No KEGG pathway was found to be significantly enriched, while the gene annotated as “homeobox protein SIX1” was found to be significantly enriched in six GO terms, including “extraocular skeletal muscle development”, “regulation of skeletal muscle cell proliferation”, and “pituitary gland development”. As for the DEGs of the NeoB-vs-CoMB group, which represented the differences between neo-male and wild-type male brains, respectively, there were only 1,585 DEGs. Though no KEGG pathway was found to be significantly enriched, 11 GO terms were significantly enriched, including “extraocular skeletal muscle development”, “tRNA (guanine-N7-)-methyltransferase activity”, and “pharyngeal muscle development”.

**FIGURE 4:**
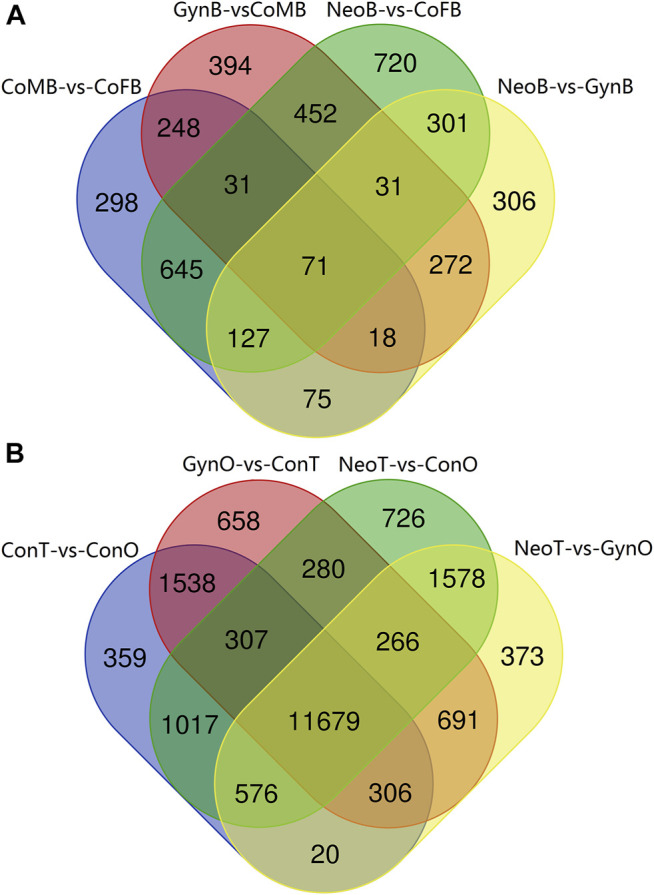
Venn diagram of the male_brain-vs-female_brain **(A)** and testis-vs-ovary **(B)** groups.

A Venn diagram of the DEGs among all male_brain-vs-female_brain groups, comprising CoMB-vs-CoFB, GynB-vs-CoMB, NeoB-vs-CoFB, and NeoB-vs-GynB, illustrated that only 71 DEGs overlapped with all four groups, whereas 298, 394, 720, and 306 DEGs were specific to the CoMB-vs-CoFB, GynB-vs-CoMB, NeoB-vs-CoFB, and NeoB-vs-GynB groups, respectively (Figure 4A). For the 71 overlapping DEGs, the GO term “NADP binding” was significantly enriched, including two genes, “glutathione reductase” and “glyceraldehyde-3-phosphate dehydrogenase”. With regard to the DEGs that were specific to the CoMB-vs-CoFB, GynB-vs-CoMB, and NeoB-vs-CoFB groups, no GO or KEGG terms were found to be significantly enriched. However, for the DEGs specific to the NeoB-vs-GynB group, the GO terms “muscle organ development”, “tissue development”, and “muscle structure development” were significantly enriched, including four, eight, and five candidate genes, respectively.

#### 3.2.2 Analysis Differentially Expressed Genes From Different Gonadal Groups

For the DEGs of the ConT-vs-ConO group, which represented the differences between wild-type male testes and female ovaries, there were 15,802 DEGs. Though no GO term was found to be significantly enriched, 14 KEGG pathways were significantly enriched, including “PPAR signaling pathway”, “Prolactin signaling pathway”, “Apoptosis”, “Tight junction”, and “Gap junction” (Supplementary Figure S2). For the DEGs between the GynO-vs-ConO group, which represented the differences between gynogenetic female and wild-type ovaries, respectively, there were 1,466 DEGs. No GO terms or KEGG terms were found to be significantly enriched. With regard to the DEGs between the NeoT-vs-ConT group, which represented the differences between neo-male and wild-type male testes, respectively, there were 1,750 DEGs. Though we did not find any significantly enriched KEGG pathway in this group, the GO term “cytoplasm”, including 72 genes, was found to be significantly enriched.

**FIGURE 5:**
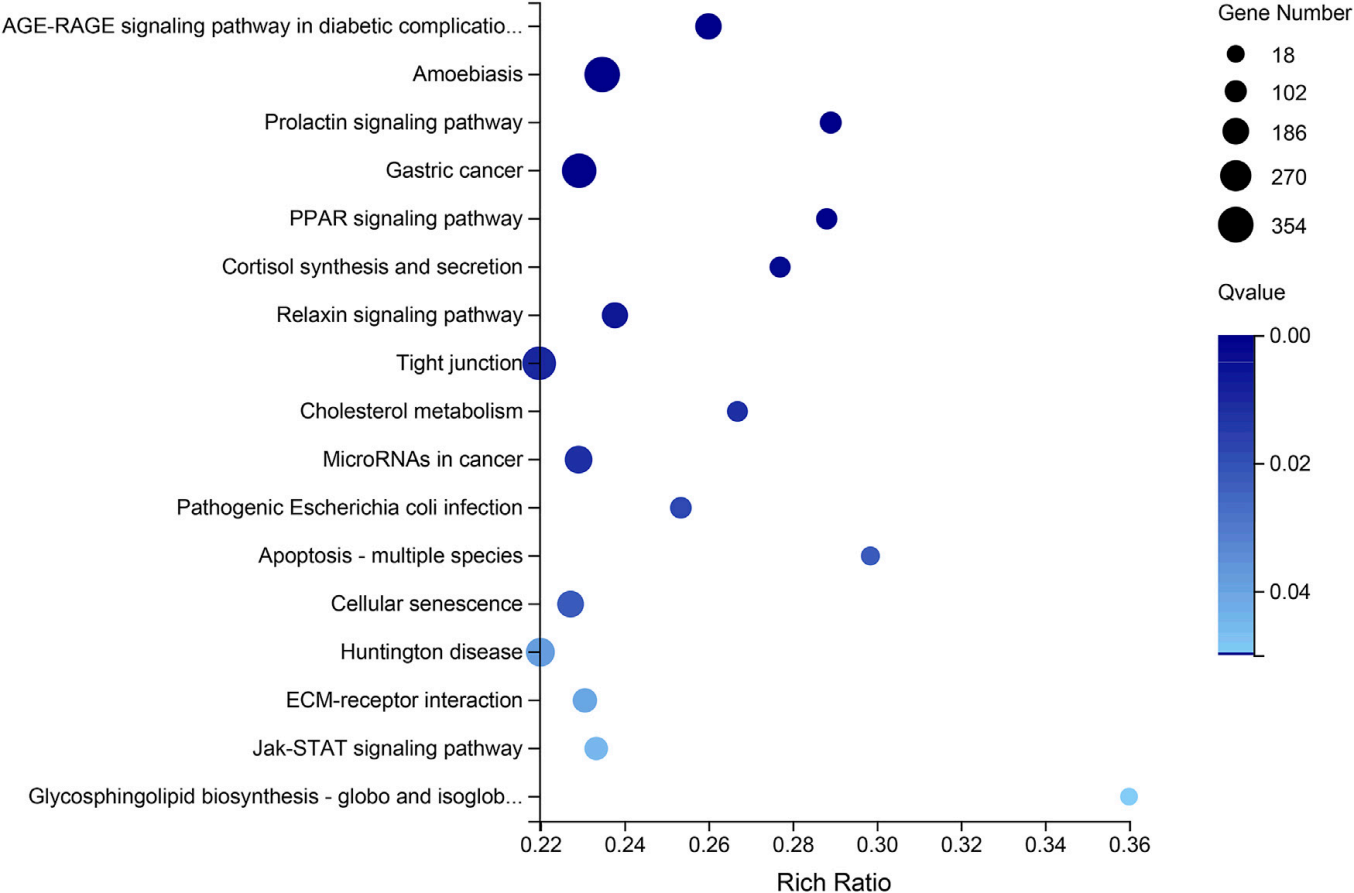
KEGG pathway enrichment based on the overlapping differentially expressed genes (DEGs) of the testis-vs-ovary group.

The Venn diagram of the DEGs among all testis-vs-ovary groups, comprising the ConT-vs-ConO, GynO-vs-ConT, NeoT-vs-ConO, and NeoT-vs-GynO groups, showed that 11,679 DEGs overlapped between the four groups, and 359, 658, 726, and 373 DEGs were specific to the ConT-vs-ConO, GynO-vs-ConT, NeoT-vs-ConO, and NeoT-vs-GynO groups, respectively (Figure 4B). For the overlapping DEGs, the GO terms “cell projection” and “plasma membrane bounded cell projection” were significantly enriched, and 17 KEGG terms were also significantly enriched, including “Prolactin signaling pathway”, “PPAR signaling pathway”, “Relaxin signaling pathway”, “Cholesterol metabolism”, and “Jak-STAT signaling pathway” (Figure 5). For the DEGs specific to the ConT-vs-ConO group, the KEGG term “Lysosome” was significantly enriched, including 13 candidate genes. For the DEGs specific to the NeoT-vs-GynO group, the GO term “tRNA (guanine-N7-)-methyltransferase activity” was significantly enriched, including two candidate genes. As for the DEGs specific to the GynO-vs-ConT or NeoT-vs-ConO group, no GO terms or KEGG terms were found to be significantly enriched.

### 3.4 Validation of RNA-Seq Data by Quantitative Real-Time PCR

**FIGURE 6:**
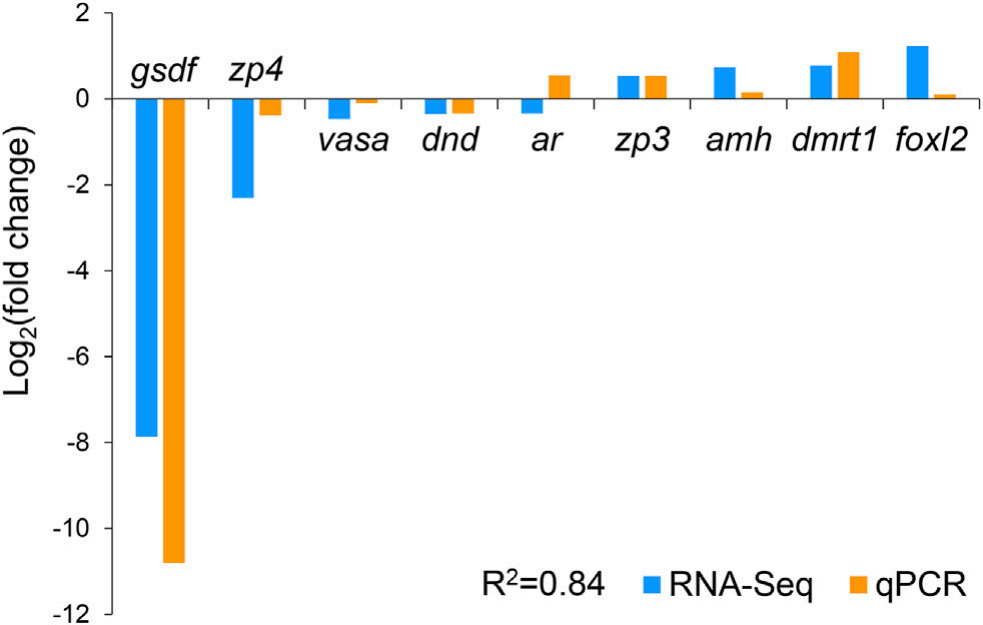
Validation of RNA sequencing (RNA-seq) data by quantitative real-time PCR (qPCR).

**FIGURE 7:**
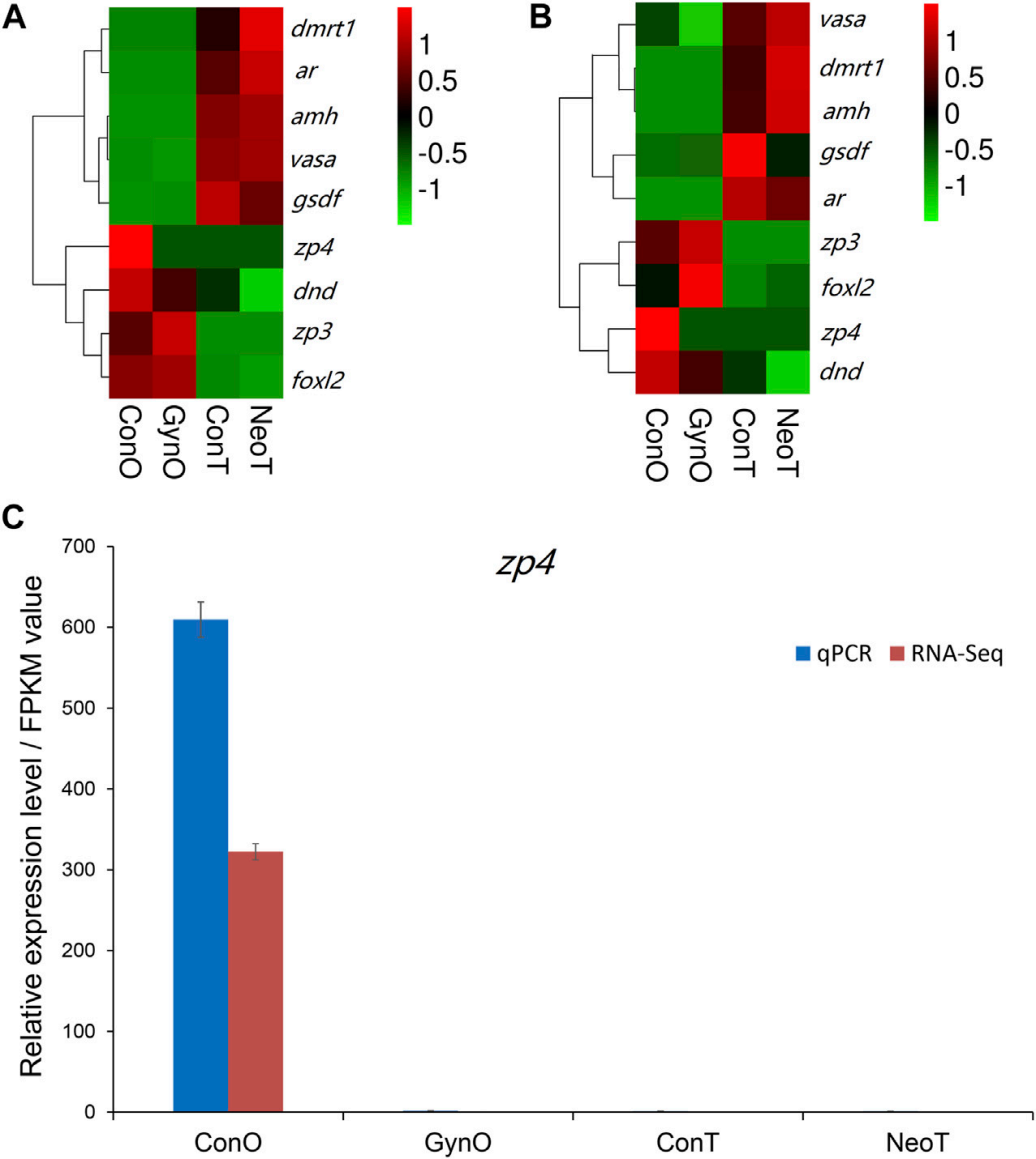
The expression patterns of the selected genes. **(A)** Heatmap of selected genes in RNA sequencing (RNA-seq). **(B)** Heatmap of selected genes in quantitative real-time PCR (qPCR). **(C)** Expression patterns/fragments per kilobase of transcript per million fragments mapped (FPKM) values of *zp4* in the gonads of different groups detected by qPCR and RNA-seq.

We selected nine DEGs for qPCR analysis, including *zp3*, *zp4*, *foxl2*, *dmrt1*, *gsdf*, *amh*, *ar*, *dnd*, and *vasa*. According to previous studies, *zp3*, *zp4*, and *foxl2* were female-related; *dmrt1*, *gsdf*, *amh*, and *ar* were male-related; and *dnd* and *vasa* were related to germ cell development. The obtained Ct values were normalized by the internal reference (*β-actin*), and the fold change was calculated accordingly (Supplementary Figure S3). As shown in Figure 6, the correlation coefficient between the Log_2_ (fold change) values of the RNA-seq and qPCR results was evaluated at 0.84, indicating a good consistency. The results of the heatmaps showing the expression patterns of the selected genes in RNA-seq and qPCR also showed good consistency (Figures 7A,B). The gene *zp4* showed significantly high expression (*p* < 0.05) only in the ovary in the control group, and its expression levels in the gonads in other groups were quite low (Figure 7C).

## 4 Discussion

In this study, to elucidate the molecular mechanisms involved in sex maintenance and gonad development in the yellow drum, we investigated the gene expression profiles in the gonads and brains of wild-type males, wild-type females, neo-males, and gynogenetic females using comparative transcriptome analysis and identified potential sex-related genes and pathways. In general, whether in the brains or the gonads, we detected low DEG numbers between wild-type males and neo-males and between wild-type females and gynogenetic females, suggesting that neo-males/gynogenetic females had transcriptomes similar to that of wild-type males/females in the brains or gonads, which was further proved by principal component analysis of the RNA-seq samples. Similar results were reported in a study of neo-male transcriptomes in zebrafish (*Danio rerio*), wherein most heat-induced sex-reversed neo-males were found to have gonadal transcriptomes similar to those of regular males (Ribas et al., 2017). The histological observation results also illustrated that neo-males and gynogenetic females exhibited normal ovarian and testicular phenotypes, respectively, compared to those of wild-type fish.

We detected relatively lower numbers of DEGs in the male_brain-vs-female_brain groups, which indicated fewer differences in the brain between sexes at the transcriptomic level. Previous studies have suggested that the brain expression profiles between sexes were different, for example, molecular changes during estrodiol-17β induced feminization were detected by RNA-seq in *Takifugu rubripes*, and genes such as *cyp19a1b*, *gnrh1*, *pgr*, *st*, *sl*, *tshβ*, *prl*, *pit-1*, etc., were found to be differentially expressed (Shen et al., 2021). Nevertheless, our result still showed many DEGs related to nervous system development were significantly enriched between male and female brains. And 71 DEGs were identified as overlapping in all four groups, and these could be potential marker genes for exploring the differences between male and female brains. In particular, three GO terms were significantly enriched in the DEGs specific to the NeoB-vs-GynB group, suggesting possible unique differences between them and potential markers for exploring these differences.

Although the number of DEGs among the testis-vs-ovary groups was relatively high, the number of overlapping DEGs in the four groups was also large (11,679 DEGs). We found that two GO terms and 17 KEGG terms were significantly enriched in this set of DEGs. Many of these pathways have been previously reported to be sex-related. For example, the Jak-STAT signaling pathway has been reported to be evolutionarily conserved and play a pivotal role during development in both vertebrates and invertebrates (Luo and Dearolf, 2001). The peroxisome proliferator-activated receptor (PPAR) signaling pathway, though seldom reported in fish, has long been reported to play a key role in estrogen regulation in mammals (Komar et al., 2001). With regard to the “Prolactin signaling pathway”, prolactin is a multifunctional polypeptide hormone and involved in many physiological processes, and it has been reported in Japanese flounder that injection of ovine prolactin could antagonize the stimulatory effect of triiodothyronine (T_3_) on the resorption of dorsal fin rays (Whittington and Wilson, 2013).

In this study, nine sex-related genes were selected for qPCR validation. *zp3*, *zp4*, and *foxl2* exhibited female-biased expression; *dmrt1*, *gsdf*, *amh*, and *ar* exhibited male-biased expression; and *dnd* and *vasa* were expressed in both gonads. Previous studies have reported that these genes are related to sex differentiation or reproductive system development (Baron et al., 2005; Rodriguez-Mari et al., 2005; Alam et al., 2008; Jin et al., 2019), which is consistent with our results. For example, *amh* was reported to be mainly expressed in Sertoli cells of the testis and granulosa cells of the ovary, and could maintain the regular proliferation and differentiation of female and male germ cells and therefore ensure the balance between proliferation and differentiation (Ijiri et al., 2008). Gene *gsdf* is the master sex determination gene in medaka (*O. luzonensis*) and Nile tilapia, where it showed male-specific high expression (Myosho et al., 2012). As one of the most conserved genes involved in sex determination and differentiation, *dmrt1* was reported to be expressed only in the mature testes of Nile tilapia (*Oreochromis niloticus*) (D. S. Wang et al., 2010) and could maintain the normal development of the testes in zebrafish (*D. rerio*) (Bradley et al., 2011). Our results also suggest that these genes play important roles in gonadal formation or function maintenance and could be used as effective molecular markers for sex identification in yellow drum. Interestingly, we noticed that similar to *zp3*, *zp4* was expressed only in the ovaries of the control group, but not in the ovaries of the gynogenetic females. In mammals, it is reported to be mainly expressed in females and plays a regulatory role in sperm–egg binding, probably as a receptor or an auxiliary receptor (Meczekalski et al., 2015). Avian *zp4* is also reported to be expressed in the oocytes, with an expression pattern similar to that of *zp2* (Serizawa et al., 2011). To date, little is known about *zp4* in teleosts, except for its female-biased expression in rock bream (*Oplegnathus fasciatus*) (Li et al., 2021). Our results showed that *zp4* might be a molecular marker to distinguish gynogenetic females from wild-type females in yellow drum. Nevertheless, a future comprehensive study is needed to investigate whether it is expressed only in wild-type and not in gynogenetic females.

In conclusion, this study identified several potential genes and pathways associated with sex maintenance and gonad development in yellow drum using transcriptional analysis. Generally, we found that the wild-type male and neo-male, and wild-type female and gynogenetic female, were quite similar on both the histological and transcriptomic levels, which could be evidence of the minimal impacts of artificial gynogenesis and hormonal sex reversal on the normal life function of female and male yellow drum, respectively. The comparative transcriptome analyses results showed that pathways such as “Oocyte meiosis”, “Cell cycle”, and “Riboflavin metabolism” were found to be significantly enriched in the brains, and those such as “Prolactin signaling pathway”, “PPAR signaling pathway”, “Cholesterol metabolism”, and “Jak-STAT signaling pathway” might play important roles in maintaining the regular proliferation of and differentiation between females and males in the gonads of the yellow drum. Several sex-biased genes, including male-biased genes, such as *dmrt1*, *gsdf*, *amh*, and *ar*, and female-biased genes, such as *zp3*, *zp4*, and *foxl2*, are potential molecular markers for the investigation of sex differentiation and sex maintenance. In particular, *zp4* might be a molecular marker for distinguishing gynogenetic females from wild-type females. The results of this study may help to elucidate the molecular mechanisms involved in sex dimorphism, sex maintenance, and sex reversal in the gonads and brains, as well as provide basic data for sex control in yellow drum breeding.

## Data Availability

The datasets presented in this study can be found in online repositories. The names of the repository/repositories and accession number(s) can be found below: National Center for Biotechnology Information (NCBI) database under BioProject accession: PRJNA821274.
