# Supplementary material for: Comparative Transcriptome Analyses Characterize Expression Signatures Among Males, Females, Neo-Males, and Gynogenetic Females in the Yellow Drum (Nibea albiflora)
Source: Front Genet. 2022 May 13;13:872815. doi: 10.3389/fgene.2022.872815 (PMC9136215; doi:10.3389/fgene.2022.872815)
Supplement: Supplementary file 1 [file DataSheet1.docx]

**Supplementary Materials**

**Table S1** Illumina sequencing and mapping statistics of the yellow drum transcriptomes.

| Sample | Total Clean Reads (M) | Total Mapping (%) | Uniquely Mapping (%) |
| --- | --- | --- | --- |
| CoFB_1 | 42.99 | 86.51 | 67.81 |
| CoFB_2 | 43.19 | 86.61 | 68.35 |
| CoFB_3 | 43.12 | 86.10 | 66.93 |
| CoMB_1 | 42.38 | 88.38 | 68.94 |
| CoMB_2 | 42.39 | 89.04 | 70.09 |
| CoMB_3 | 42.46 | 88.18 | 68.05 |
| ConO_1 | 42.60 | 86.57 | 66.85 |
| ConO_2 | 42.90 | 86.30 | 67.01 |
| ConO_3 | 43.01 | 86.87 | 67.48 |
| ConT_1 | 42.93 | 85.33 | 64.34 |
| ConT_2 | 42.96 | 84.91 | 65.27 |
| ConT_3 | 42.98 | 84.95 | 63.86 |
| GynB_1 | 42.38 | 88.95 | 70.59 |
| GynB_2 | 42.43 | 88.42 | 71.18 |
| GynB_3 | 42.47 | 88.83 | 69.99 |
| GynO_1 | 42.29 | 87.92 | 68.09 |
| GynO_2 | 42.61 | 87.6 | 68.02 |
| GynO_3 | 42.45 | 87.59 | 68.03 |
| NeoB_1 | 43.07 | 86.28 | 63.66 |
| NeoB_2 | 42.19 | 86.04 | 63.43 |
| NeoB_3 | 43.24 | 87.84 | 66.40 |
| NeoT_1 | 43.05 | 86.1 | 66.65 |
| NeoT_2 | 42.95 | 85.22 | 63.73 |
| NeoT_3 | 43.05 | 86.40 | 66.03 |
| Average | 42.75 | 86.96 | 67.12 |

**Table S2** Detailed information on the primers used in the qPCR validation.

| Gene annotation | Gene abbreviation | Primers (5’-3’) |
| --- | --- | --- |
| Doublesex and mab-3 related transcription factor 1 | *dmrt1* | -F AAACCACGGCTATGTCTCAC |
|  |  | -R CGTCCTTCCATAGAGTACAAACAG |
| Zona pellucida sperm-binding protein 3 | *zp3* | -F CTGGGCCTCAAGCTGATAAA |
|  |  | -R GCTGTGCAGCTCATATTCAAAG |
| Zona pellucida sperm-binding protein 4 | *zp4* | -F GGGTTACGTGGTGTATGAGAAC |
|  |  | -R GCAGAATAGCGACACTGGAATA |
| Gonadal soma-derived factor | *gsdf* | -F CAACTGGGTGATCCATCCTAAA |
|  |  | -R CGCTGAATTCATCCACAAAGAC |
| Dead end | *dnd* | -F GTCTGCACTGGTCACATCAT |
|  |  | -R TCAGGTGGTCCTCCATACTT |
| Vasa | *vasa* | -F GTGTGCGAAAACGGCTTTAG |
|  |  | -R CCATCGCTTCCATCCTTATTTTC |
| Anti-Müllerian hormone receptor | *amh* | -F TCTTGCTCAACTCCCACATC |
|  |  | -R CGTAGCCACCACGTCTATTT |
| Androgen receptor | *ar* | -F CACTGGGATGGAGGTCCTATAA |
|  |  | -R TGCTCGTACATGGTGGAAATG |
| Forkhead box L2 | *foxl2* | -F GGAGAAAGAGCGACCTAAAGAG |
|  |  | -R TGGCAATGAGAGCGACATAAG |
| β-actin | *β-actin* | -F CCTCCCTGGAGAAGAGCTATGAG |
|  |  | -R CGCACTTCATGATGCTGTTGTAG |


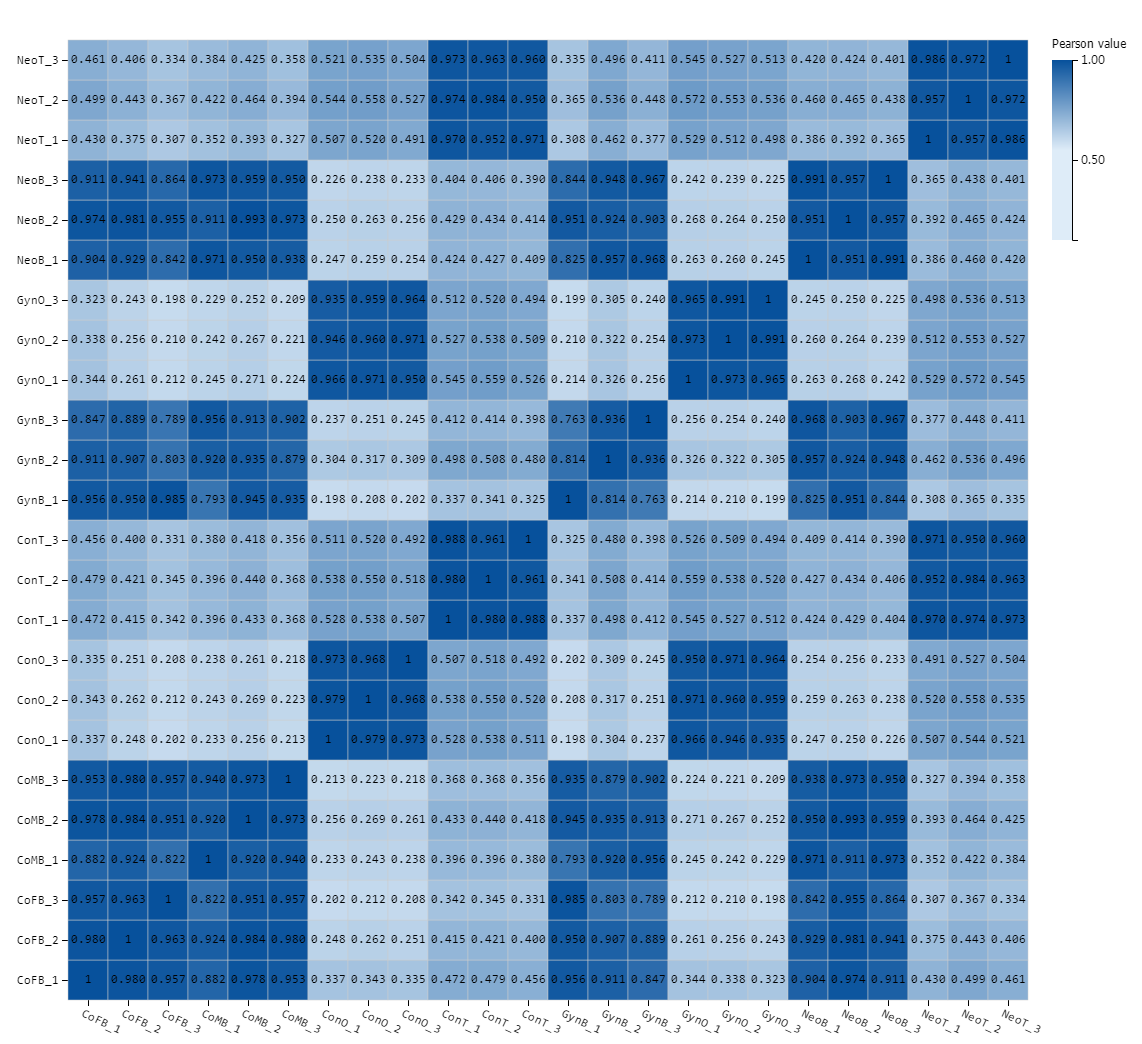


**Fig. S1** The correlation coefficients between RNA-Seq samples.


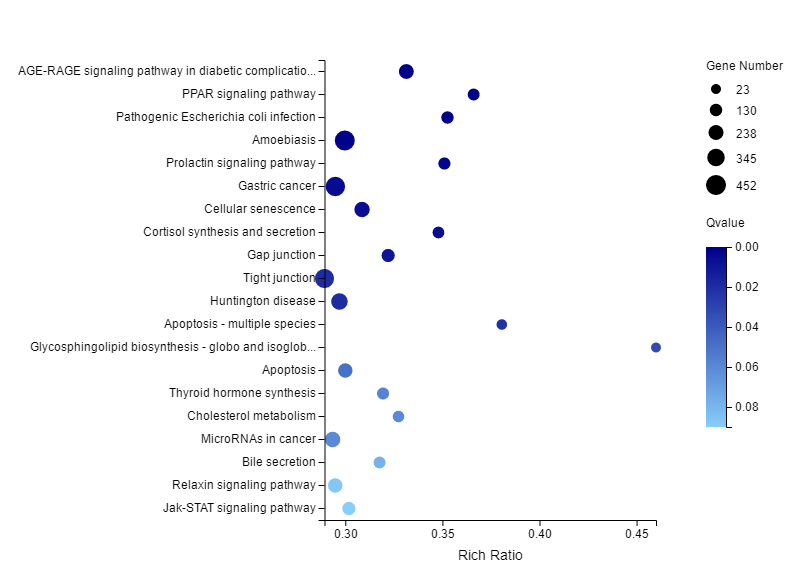


**Fig. S2** KEGG pathway enrichment based on the DEGs of the ConT-vs-ConO group.


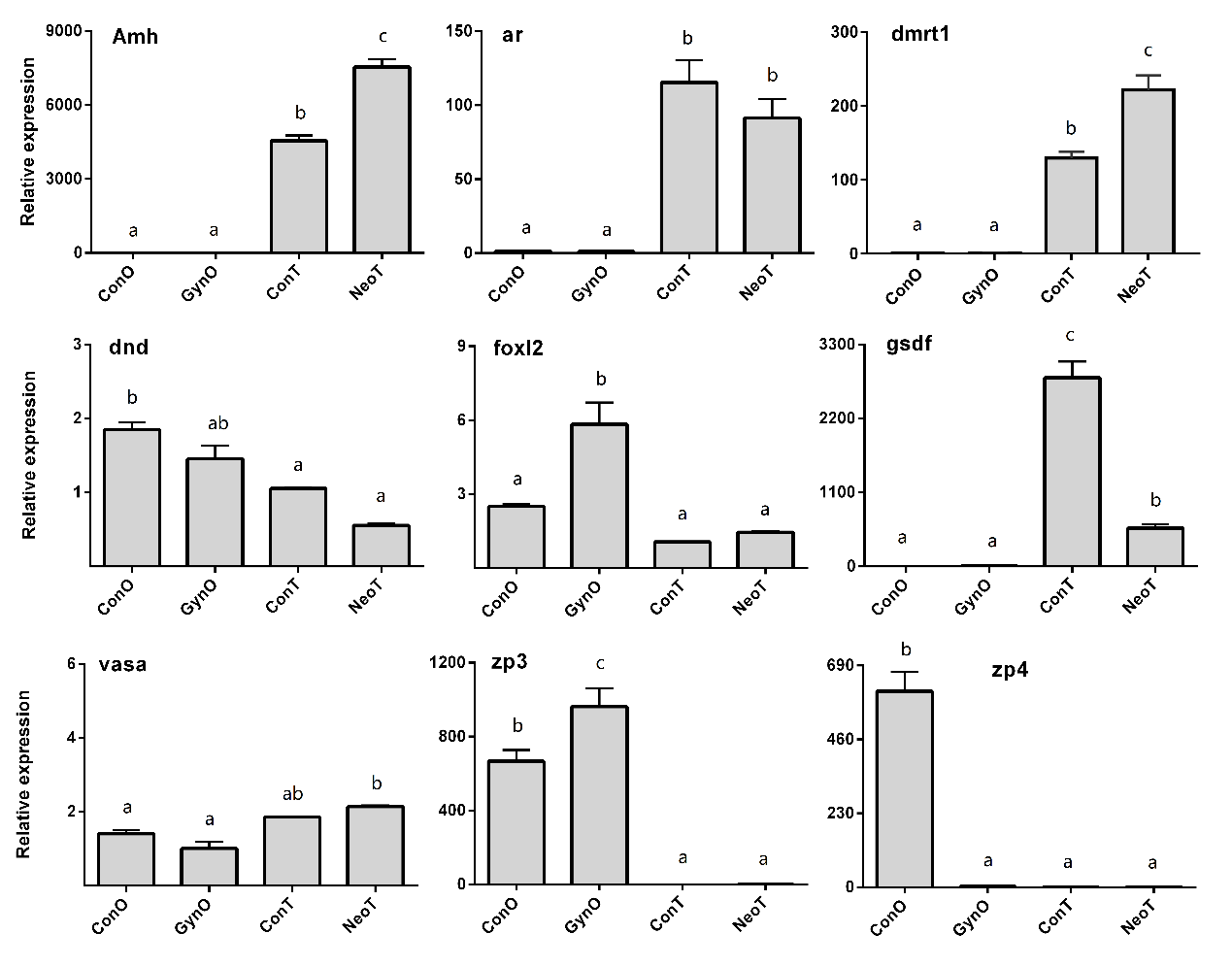


**Fig. S3** The detailed result of qPCR validation of all nine genes.
